# Determinants for return to work among sickness certified patients in general practice

**DOI:** 10.1186/1471-2458-12-1077

**Published:** 2012-12-14

**Authors:** Anna-Sophia von Celsing, Kurt Svärdsudd, Hans-G Eriksson, Karin Björkegren, Margaretha Eriksson, Thorne Wallman

**Affiliations:** 1 Department of Public Health and Caring Sciences, Family Medicine and Preventive Medicine Section, Uppsala University, Uppsala, Sweden; 2 Centre for Clinical Research Sörmland, Uppsala University, Eskilstuna, Sweden

## Abstract

**Background:**

Long-term sickness absence is one of the main risk factors for permanent exit out of the labour market. Early identification of the condition is essential to facilitate return to work. The aim of this study was to analyse possible determinants of return to work and their relative impact.

**Methods:**

All 943 subjects aged 18 to 63 years, sickness certified at a Primary Health Care Centre in Sweden from 1 January until 31 August 2004, were followed up for three years. Baseline information on sex, age, sick leave diagnosis, employment status, extent of sick leave, and sickness absence during the year before baseline was obtained, as was information on all compensated days of sick leave, disability pension and death during follow-up.

**Results:**

Slightly more than half the subjects were women, mean age was 39 years. Half of the study population returned to work within 14 days after baseline, and after three years only 15 subjects were still on sick leave. In multivariate proportional hazards regression analysis the extent of previous sick leave, age, being on part-time sick leave, and having a psychiatric, musculoskeletal, cardiovascular, nervous disease, digestive system, or injury or poisoning diagnosis decreased the return to work rate, while being employed increased it. Marital status, sex, being born in Sweden, citizenship, and annual salary had no influence. In logistic regression analyses across follow-up time these variables altogether explained 88-90% of return to work variation.

**Conclusions:**

Return to work was positively or negatively associated by a number of variables easily accessible in the GP’s office. Track record data in the form of previous sick leave was the most influential variable.

## Background

Disability pension has been shown to be associated with negative health development [1-5]. Long-term sickness absence is one of the main risk factors for permanent exit out of the labour market. There is no generally established definition of long-term sickness absence in the literature, and number of days, regarded as long-term sickness absence, vary considerably between different studies i.e. from >3 days to 90 days or more [6,7]. The reason for choosing a specific cut-off level of days can be the availability of sickness absence data [8] or adjustment to the current social insurance legislation [9]. Long-term sickness absence usually begins with recurrent periods of short-term sick leave that tend to increase in duration, interspersed with shorter and shorter non-sick leave intervals [10]. Long-term sickness absence may also be expressed in terms of return to work, since long-term sickness absence is inversely related to return-to-work, when long-term sickness decreases return to work increases, all things considered.

Early detection of patients at risk for long-term sickness absence may be of importance for identification of individuals in need of rehabilitation measures in order to regain their work ability [11]. A number of potential determinants have been identified such as female sex, old age, low socio-economic status, and previous spells of sick leave [12-15]. Some sick leave diagnoses appear to be more prone to long-term sick leave periods than others, such as musculoskeletal disorders, psychiatric disease, and cardiovascular disorders [1,7,16-24]. Work-related factors, such as physically heavy work, high work demands, low work control, low job satisfaction, relational problems at the workplace, and a stressful work situation tend to increase long-term sickness absence [9,25,26] and thereby postpone return to work, as does being unemployed [27] and being on part-time sick leave [28].

All Swedish permanent residents, whether citizens or not, have a unique 12-digit personal identification number (PIN), given at birth or immigration and used in all official documents and registers. The PIN is an excellent and highly reliable tool for record linkage. Moreover, all residents are covered by the National Social Insurance, which includes the right to see a physician of ones own choice and access to hospital care at heavily subsidized rates, to have sickness benefit for income loss in case of reduced work capacity due to injury or disease, and a number of other items. The National Social Insurance is regulated by the National Insurance Act and is managed by the National Social Insurance Agency (SIA), a government agent with offices in all municipalities across the country.

Sick listing is a common and problematic task for general practitioners (GPs) [29-32]. One problem is the assessment as to whether a patient will return to work or not after a period of sick leave. In general practice, there is seldom time during a brief consultation for deep sickness history penetration, or for time-consuming identification of risk factors for long-term sickness absence. However, even when identified the relative impacts of long-term sickness absence determinants on return to work are not well known.

County Councils run the overwhelming part of Swedish medical care. They are responsible for health care within their area; either at County Council operated primary health care centres, at the time of the study the vast majority, or at subcontracted private primary health care centres. However, all centres, whether County Council operated or privately subcontracted, follow the same regulations.

In case of sick leave, patient’s self-certification is accepted for an initial period of seven days. After this period a sickness certificate form has to be completed by the patient’s physician and sent to the patient’s work place. At the time of data collection the employer was responsible for sickness compensation during the first 14 days. If the sickness absence persisted after this time, the sick leave certificate was sent to the local SIA office, which then took responsibility for sickness compensation and for further handling. For unemployed subjects SIA took responsibility for sickness compensation from day 1. Otherwise, the same procedure as for employed subjects was followed.

Information on age, sex, occupational status, sick leave diagnoses, examination results, impaired functions attributable to the disease causing the reduced working capacity, suggested degree of sick leave, and suggestions for various rehabilitation measures to regain work capacity have to be documented in the certificate to enable a decision by SIA as to whether the patient fulfils the criteria for further sickness compensation.

The aim of this study was to analyse possible determinants of return to work and their relative impacts, in order to arrive at a simple model by which return to work might be estimated early in the sick leave process.

## Methods

### Setting

The study was performed in the city of Eskilstuna, Sweden, at one of the County Council operated primary health care centres, with ten GPs serving a population of approximately 25,000 residents. Eskilstuna is an industrial city with 91,000 residents in 2004, located 110 kilometres west of Stockholm.

### Study population

The study was designed as a three-year prospective cohort study with recruitment from 1 January until 31 August 2004. During the recruitment period copies of all sickness certificates, whether new or prolongation certificates, issued at the primary health care centre, were obtained. All individuals aged 18 to 63, who were sickness certified by a physician at the centre at any time during the recruitment period, and who were not already included in a medical or vocational rehabilitation programme, were included, 482 women and 461 men, altogether 943 subjects.

Information on sex, attained age at the baseline examination, being born in Sweden, citizenship, marital status (classified as never married, married, divorced or widowed), occupational status (being employed or not), salary at baseline expressed in Euro, sick leave diagnosis, and degree of sick leave (25%, 50%, 75% or 100%) was obtained from the local SIA office. Information on sickness absence during the year preceding the baseline examination and the three years following baseline was obtained from the SIA database, and also from the primary health care medical records in order to have valid information on sick leave during the first 14 days.

Outcome in this study was conclusion of the sick certification period in effect at baseline, in other words return to work, although some subjects did not have a work to return to. Data on all certified sick leave periods for the three years following baseline, including first and last day of each sickness spell, type of sickness benefit (compensation for sickness or rehabilitation), sick leave diagnosis, degree of sick leave, and whether a disability pension was granted during follow-up, was obtained from the SIA national database. Information on vital status was obtained from the National Cause of Death Register, providing date of death for those who died (n = 6). The sick leave diagnoses were coded according to the International Classification of Diseases (ICD-10) [33]. The Regional Ethics Review Board, Stockholm, Sweden, approved the study.

### Statistical methods

Data were analysed with the SAS software, version 9.2. There was no missing data. The three-year sick leave follow-up data were converted into a day-by day matrix starting with variable day 0 (baseline day) and ending with variable day 1095 (end of follow-up), each variable measuring whether the subject was on sick leave (=1) or not (=0) on that day.

Based on this matrix a return-to-work variable was computed. For each sick spell the following two return-to-work criteria were applied. Criterion 1: the sick spell was followed by a sick leave free interval of more than 28 days, regardless of the length of any following sick spell. Criterion 2: the sick spell was followed by a sick leave free interval of more than 7 days, and that interval was longer than the next sick spell. When at least one of the criteria was satisfied, return to work was presumed to have occurred on the first non-sick leave day. If none of the criteria were satisfied at end of follow up no return-to-work was presumed to have occurred.

Follow-up time from baseline to return to work or end of follow-up was measured as number of days from baseline. Determinants for return to work were tested with proportional hazards regression technique (Cox’s analysis), using the SAS procedure ‘Phreg’, with return to work and the day when this occurred as outcome, and age at baseline, sex, number of days of sick leave during the year preceding baseline, whether on full time or part time sick leave, marital status, whether employed, born in Sweden, being a Swedish citizen or not, salary during the last year, and sick leave diagnosis included as potential determinants. The procedure provides hazards ratios (HR), 95% confidence intervals (95%CI), and Wald’s chi-square, the latter, being the test parameter and computed with one degree of freedom for all variables regardless of grading, and therefore used as measure of determinant impact on outcome.

The analyses were performed in two steps. First, orienting bivariate analyses were performed, one for each potential determinant, followed by multivariate analyses. In the latter, variables with mutually exclusive responses, such as marital status and sick leave diagnoses, were analysed with dummy variables. For marital status, being married, which had the hazard ratio (HR) closest to 1, was chosen as reference for the effect of marital status, and regarding sick leave diagnoses, respiratory system disease (ICD-10 code J, (in most cases upper respiratory tract infections) had the same characteristics and was used as reference for the effects of the diagnoses. To arrive at interpretable HRs, number of days of sick leave during the past year was recomputed as number of weeks, and age as five-year age groups.

The analyses were performed straightforwardly with sex as determinant as well as stratified for sex. In the latter, the results for women and men were similar. For this reason only results from the former type of analysis are shown.

**Figure 1:**
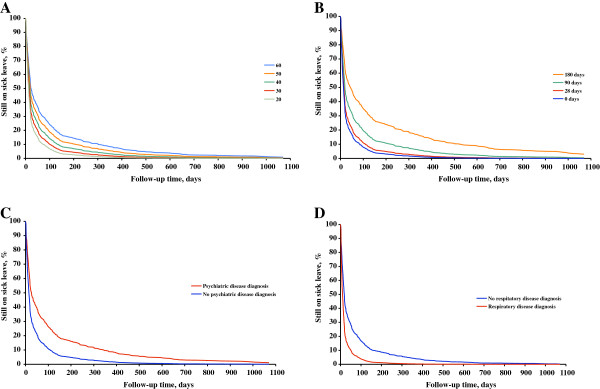
**Return to work.** Return to work during three years of follow-up in groups according to age (**A**), number of sick leave days the year before baseline (**B**), sick leave for to psychiatric diagnosis (**C**), and sick leave for to respiratory disease diagnosis (**D**).

The content of Figure 1 was computed with proportional hazards regression technique using the same multivariate analysis model as for the multivariate analyses. Data on the effects on return-to-work of age, sick leave days during the year before baseline, having a psychiatric diagnosis, a having a respiratory disease variable was obtained from the analysis model. Regarding age data for persons aged 20, 30, 40, 50, or 60 years, and for sick leave during the year before baseline days 0, 28, 90, and 180 were obtained from the model. Regarding the diagnoses data on those with a diagnosis and those with no such diagnosis were obtained from the analysis model.

The degree of explanation, *i.e.* how well the determinants could explain return to work, was measured with nominal logistic regression across the follow-up period during the first 7, 14, 28, 90, 180, 270, 365, 455, and 545 days, using Concordance index (C), an estimate of the area under the receiver operating curve characteristic (ROC) curve, and a standard option in SAS ‘Logist’ procedure, and with the same variable set up as in the proportional hazards regression analysis model [34]. All tests were two-tailed and the significance level was set at p < 0.05.

## Results

### Characteristics of the study population

Slightly more than half the subjects were women, mean and median age were 39 years, more than 75% were employed, more than 93% were Swedish citizens, 44% were never married and somewhat more than one third were married, mean annual income was 22,000€, mean number of days of sick leave during the year preceding baseline was 51, and the vast majority were on full-time sick leave, Table 1. The most common sickness certification diagnoses in rank order were musculoskeletal disease, psychiatric disease, and respiratory system disease.

**Table 1:** Characteristics of the study population

	**Women**		**Men**	
	**n**	**Mean (SD) or%**	**n**	**Mean (SD) or%**
Age, years				
Mean	482	39.1 (12.04)	461	39.2 (11.66)
Inter-quartile range		29-49		30-48
Employed,%	365	75.7	356	77.2
Born in Sweden,%	389	80.7	342	74.2
Swedish citizen,%	454	94.2	429	93.1
Marital status,%				
Never married	195	40.5	218	47.3
Married or cohabiting	183	38.0	162	35.1
Divorced	90	18.7	79	17.1
Widowed	12	2.5	2	0.4
Annual salary at baseline, €				
Mean	373	20,127 (5,386)	342	24,241 (8,195)
Inter-quartile range		17,333-23,333		20,000-26,667
Sick leave before baseline, days				
Mean		55.1 (105.82)		48.8 (94.69)
Inter-quartile range		0-49		0-35
Full-time sick leave at baseline	431	89.4	430	93.3
Sick leave diagnoses,%				
Musculoskeletal disease	150	31.1	152	33.0
Psychiatric disease	131	27.2	90	19.5
Respiratory disease	88	18.3	70	15.2
Injury, poisoning	17	3.5	34	7.4
Symptoms and signs	33	6.9	31	6.7
Infectious-parasite disease	12	2.5	20	4.3
Dermatology disease	9	1.9	9	2.0
Cardiovascular disease	8	1.7	10	2.2
Genitourinary system disease	7	1.5	8	1.7
Digestive system disease	5	1.0	13	2.8
Eye or ear disease	5	1.0	6	1.3
Nervous system disease	5	1.0	5	1.1
Endocrine-metabolic disease	4	0.8	3	0.7
Blood disease	3	0.6	1	0.2
Pregnancy, childbirth	1	0.2	0	-
Miscellaneous	4	0.8	9	2.0

Baseline characteristics of the study population.

At baseline all subjects were on sick leave. Among both men and women 50% of the subjects had returned to work within 14 days, 75% of the men within 55 days and 75% of the women within 80 days. At end of follow-up 6 (1.3%) men and 9 (1.9%) women were still on sick leave.

### Potential determinants of return to work

The effects of potential determinants for return to work are shown in Table 2. In bivariate analyses previous sick leave, age, present part-time sick leave, being divorced, being born in Sweden, and being a Swedish citizen all decreased the probability of return to work, while male sex, being never married, and being employed increased it. Annual salary had no significant influence.

**Table 2:** Return to work determinants

	**Bivariate analysis**				**Multivariate analysis**			
	**HR**	**95%CI**	**Wald’s χ**^**2**^	**p**	**HR**	**95%CI**	**Wald’s χ**^**2**^	**p**
Previous sick leave by week	0.97	0.96-0.97	150.5	<0.0001	0.97	0.97-0.98	92.0	<0.0001
Age by 5-year groups	0.91	0.88-0.94	38.6	<0.0001	0.95	0.92-0.99	6.9	<0.01
Employed	1.38	1.18-1.62	16.3	<0.0001	1.28	1.02-1.59	4.7	<0.05
Part-time sick leave	0.57	0.45-0.72	22.4	<0.0001	0.75	0.58-0.98	4.5	<0.05
Marital status								
married	1.10	0.96-1.25	1.8	0.17	1.00	reference	-	-
never married	1.16	1.02-1.32	5.1	<0.05			2.3	0.13
divorced	0.74	0.62-0.87	12.7	<0.0005			0.9	0.33
widowed	0.61	0.35-1.06	3.1	0.08			0.3	0.56
Born in Sweden	0.82	0.70-0.95	6.6	<0.05			1.5	0.22
Male sex	1.16	1.02-1.32	4.7	<0.05			1.3	0.26
Annual salary by 10,000 €	0.99	0.90-1.08	0.1	0.80			0.7	0.39
Swedish citizenship	0.77	0.59-0.99	4.0	<0.05			0.03	0.85
Diagnosis								
Respiratory disease	2.36	1.97-2.82	88.3	<0.0001	1.00	reference	-	-
Psychiatric disease	0.70	0.60-0.82	20.6	<0.0001	0.31	0.25-0.40	94.9	<0.0001
Musculoskeletal disease	0.79	0.69-0.91	11.2	<0.001	0.41	0.33-0.51	67.6	<0.0001
Cardiovascular disease	0.72	0.45-1.14	1.9	0.16	0.30	0.17-0.52	18.4	<0.0001
Symptoms and signs	1.33	1.03-1.71	4.7	<0.05	0.53	0.38-0.75	12.8	<0.0005
Nervous system disease	0.68	0.35-1.32	1.3	0.26	0.26	0.12-0.57	11.6	<0.001
Digestive system disease	0.82	0.51-1.32	0.7	0.41	0.41	0.24-0.71	10.1	<0.005
Injury, poisoning	1.12	0.84-1.49	0.6	0.44	0.56	0.39-0.80	9.9	<0.005
Miscellaneous	1.02	0.59-1.77	0	0.93	0.48	0.23-0.98	4.1	<0.05
Endocrine-metabolic disease	1.29	0.61-2.71	0.4	0.50			3.0	0.08
Eye or ear disease	1.83	1.01-3.31	3.9	<0.05			2.8	0.09
Infectious-parasite disease	1.62	1.14-2.31	7.2	<0.001			2.2	0.14
Genitourinary system disease	1.21	0.73-2.02	0.6	0.46			1.5	0.22
Blood disease	0.72	0.27-1.91	0.4	0.50			1.0	0.32
Dermatology disease	1.63	1.02-2.61	4.2	<0.05			0.6	0.43
Pregnancy, childbirth	5.53	0.77-39.52	2.9	0.09			-	-

Potential determinants of return to work within the next 1095 days.

Among the diagnoses some positively and other negatively associated with the probability of return to work. The most influential ones were respiratory system disease, infectious disease, eye or ear disease, and unspecific symptoms and signs that increased probability, while psychiatric and musculoskeletal disorders decreased the probability of return to work.

In multivariate analysis previous sick leave, age, present part-time sick leave, and being employed kept their significance for return to work, while marital status, male sex, being born in Sweden, citizenship, and annual salary did not. Among the diagnoses psychiatric and musculoskeletal disorders, cardiovascular diseases, unspecific symptoms and signs, nervous system disease, digestive system disease, injury and poisoning, and miscellaneous diseases were significantly associated with decreased probability for return to work.

The effects over time of age, previous sick leave, having a psychiatric diagnosis and having a respiratory system diagnosis are shown in Figure 1. For each 10 years of age from age 30 the return to work was slower than in the previous decade. On the 100th day 7% of the 20-year olds were still on sick leave versus 25% of the 60-year old. Among those who had no sick leave days during the last year 8% were still on sick leave on the 100th day versus 35% of those with 180 sick leave days during the last year. Among those with no psychiatric diagnosis 11% were on sick leave on the 100th day versus 27% among those with such a diagnosis, and finally among those with an upper respiratory tract diagnosis 4% were still on sick leave on the 100th day as compared with 17% among those with other diagnoses.

### Degree of explanation

**Figure 2:**
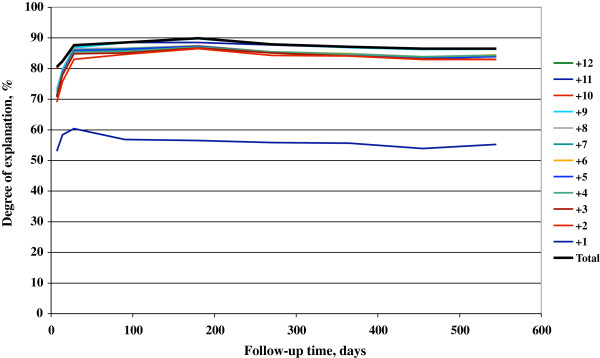
**Degree of explanation.** Cumulative degree of explanation of twelve significant return-to-work determinants, given in ranking order according to importance (Wald’s χ2) and measured on days 7, 14, 28, 90, 180, 270, 365, 455, and 545. 1 = sick certified for psychiatric disease, 2 = number of sick leave days the year before baseline, 3 = sick certified for musculo-skeletal disease, 4 = sick certified for cardiovascular disease, 5 = sick certified due to unspecified symptoms or signs, 6 = sick certified for nervous system disease, 7 = sick certified for digestive system disease, 8 = sick certified for injury or poisoning, 9 = age at baseline, 10 = employed, 11 = extent of sick leave, 12 = sick certified for miscellaneous disease.

The degree of explanation across the first one and a half year of follow-up is shown in Figure 2. Using only the most influential variable, sick leave due to psychiatric diagnosis, the degree of explanation would be approximately 55% throughout the follow-up period. Including also the previous sick leave track record increased the degree of explanation to approximately 85%, and by including all other significant determinants degree of explanation approached 88% -90%.

## Discussion

The degree of explanation for all variables combined was slightly short of 90%, indicating that the major influencing variables were included in the analysis model. The two most influential variables, sick leave because of psychiatric disease and sick leave track record together explained approximately 85% of return to work. The determinants of return to work in this study in rank order were sick leave because of psychiatric disease, sick leave track record, sick leave because of musculoskeletal disease, cardiovascular disease, symptoms and signs, nervous system disease, digestive system disease, injuries and poisoning, and age, being employed, extent of sick leave and miscellaneous diseases. The latter four variables had only marginal effects.

Return to work was in this study defined as termination of the sick leave period according to the criteria used, even though all study participants may not have had a work to go back to. We know which of the participants that were out of a job at baseline, but we do not know the situation at the time when the sick period was terminated. They may have gone back to work, or been granted a disability pension, or been granted social welfare, or been able to support themselves in other ways. Return to work should therefore in this context be interpreted as leaving the sick leave status.

The effects on long-term sickness absence (or return to work) of female sex, increasing age and unemployment have previously been reported in other studies [10,14]. In the present study sex was moderately influential in bivariate with a Wald’s chi-square of 4.7 as compared to previous sick leave that had a Wald’s chi-square of 150.5. It is therefore not surprising that sex had no significant influence in multivariate analysis. It was simply competed out by variables with much stronger impact. Performing the analyses with sex as a determinant or stratified according to sex made no difference. The effects of some of the sick leave diagnoses, especially psychiatric disease and musculoskeletal disorders that tend to prolong sick leave, have previously been shown [16,18,19,21,24,35,36]. In this study a number of other diagnoses proved to be significant determinants, some facilitating, others delaying return to work, a novel finding, not reported before.

In the present study information on previous sick leave, or sick leave track record, was one of the most informative factors for predicting return to work. The possible use of administrative sickness absence data as a risk marker of future sickness absence and disability pension was demonstrated in a Danish study [37]. These results have been confirmed in Dutch and British studies [38,39]. Previous sick leave as indicator for chronic health problems was shown in a large-scale cohort study [40]*.* In 1995, Marmot in a study investigating the relationship between self reported health status and sickness absence, found a strong association between ill health and sickness absence, particularly for longer spells [9]. Moreover, in a previous publication from this project previous sick leave, or sick leave track record, was closely associated with being granted a disability pension [10].

Are there more determinants for return-to-work, not measured in this study? Yes, probably, but these potential determinants are unlikely to change the main results of this study. Even if such a determinant has a strong impact on return-to-work in bivariate analysis, the potential impact in the multivariate analysis model used in this study is at the most 10%, since the variables included explained 90% of return-to-work. This means that potential determinants not included in the study most probably have a marginal effect as compared to variables included in the present study.

In the UK, GPs often consider the judging of whether or not a patient is fit for work, to be a “highly complex process, involving reconsideration, uncertainty, and a number of stages and types of deliberation” [41]. In a survey among Swedish physicians in two counties [42] a larger proportion of physicians at orthopaedic clinics and, in particular, at primary health care centres, experienced more sick-listing problems than physicians at other clinics. Engblom *et al.*[43] identified categories of specific difficulties GPs experience in their clinical practice when face-to-face with the patient.

Sick-listing difficulties appear to be shared by physicians in several countries [44-47]. Reiso *et al.*[48] raised the question of how well a GP may predict return to work, and found GPs’ predictions for return to work highly accurate for short-term episodes but less so for more long-term ones. In a Norwegian study, sick-listed individuals predicted their length of sick leave more accurately than professionals [49].

The strengths of this study include that the study population was large enough for the purpose of the study, that it was based on all patients passing through the ‘time window’ of data collection, making it equivalent to a random patient sample. The exposure and outcome data used in the analyses were obtained from the official SIA sick leave database, and from medical records regarding the first two weeks of sick leave. Data was complete with no data loss.

One possible limitation of the study could be the choice of the six determinants (counting diagnoses as one determinant), as other variables might serve the same purpose for screening individuals at risk for long-term sickness absence. However, the variables chosen have been shown to be long-term sickness absence determinants in previous studies [14], and five of the six are readily available to the GP when issuing a sickness certificate. The sixth variable, previous periods of sick leave, is usually accessible information in the primary health care records. At any rate, the variables chosen proved to be quite sufficient for the prediction purpose. The 10% unexplained return to work proportion indicates that no major determinant was overlooked.

The implications of the result of the present study might be that the existing difficulties in assessing the possibilities for return to work are relatively easily overcome, since the number of variables to take into account is limited. As shown in Figure 2, access to all six variables provided an excellent degree of explanation. However, access to only the two most powerful variables, sick leave diagnosis and sick leave track record during the past year, yielded an explanation of return to work of approximately 85% as early as during the first month. Sick leave diagnosis is always available. Similarly, sick leave track record is readily available in countries with a GP gatekeeping system, while GPs in other health care systems may have to rely on information given by patients. A better availability for the GP of sick leave track record from the stakeholders should improve the possibilities to arrive at a reasonably precise sick leave prognosis.

## Conclusions

A number of variables were associated with return to work. Track record data in the form of previous sick leave was the most influential variable. Together the determinants explained 88-90% of the return to work variation during follow-up. However, the two most important determinants together explained approximately 85% of the return to work. It might therefore be possible to assess the possibilities of return to work based on data available at the time of sick certification, thereby avoiding long-term sickness absence.

## Competing interests

The authors declare that they have no competing interests.

## Authors’ contributions

ASvC was responsible for the conception and design of the study and data collection. HE compiled the database. HE and KS analysed the data. ASvC, TW and KS drafted the manuscript. All authors participated in the interpretation of the results, the revision of the manuscript, and the approval of the final manuscript version.

## Pre-publication history

The pre-publication history for this paper can be accessed here:

http://www.biomedcentral.com/1471-2458/12/1077/prepub
